# The challenges imposed by COVID-19 on the management of diagnostic centers

**DOI:** 10.1590/0100-3984.2020.0160

**Published:** 2021

**Authors:** Christiano Berti, Bruno Hochhegger

**Affiliations:** 1 MV Sistemas, Porto Alegre, RS, Brazil.; 2 Departamento de Radiologia, Universidade Federal de Ciências da Saúde de Porto Alegre (UFCSPA), Porto Alegre, RS, Brazil.

## INTRODUCTION

The coronavirus disease 2019 (COVID-19) pandemic has profoundly affected businesses in all sectors, the health care sector being one of many that have faced even more challenging developments. In addition to the prevention and treatment of the disease itself, there is also the issue of business: hospitals and other health care facilities face high daily variation in patient demand, as well as administrative changes. The management of diagnostic centers was also affected, not only because they have been at the forefront of the diagnosis of COVID-19 but also because of the drop in demand for other types of tests. Therefore, we are facing an unprecedented situation, the consequences of which we cannot yet predict, although some of them are already beginning to take form. In view of that, we present here six challenges that should be borne in mind for the management of diagnostic centers.

### Reduction in the demand for elective procedures

In Brazil, we see a scenario similar to that experienced in the United States: there were reports of substantial drops in the demand for diagnostic imaging services across the country, reaching a 56.4-63.7% reduction among inpatients, as shown in a study published in August of 2020 in the Journal of the American College of Radiology^(1)^. The overall drop in the number of tests performed in Brazil was even greater, reaching 80% in February and March of 2020, according to figures published by the Brazilian National Association of Private Hospitals^(2)^. Data from the Brazilian Association of Diagnostic Medicine show a similar trend: diagnostic imaging clinics registered a 70% drop in demand in comparison with 2019^(2)^. There was also a 60% reduction in the demand for the services offered by clinical laboratories. Despite those numbers, demand remained high at many facilities that work with urgent cases, due to the nature of COVID-19, which requires examinations such as X-ray and computed tomography for the diagnosis of the disease, as well as for monitoring its progression and the involvement of the lungs. In that context, diagnostic centers that had already adopted a picture archiving and communication system had an advantage, because that technology promotes agility and increases the ability to visualize the affected areas, serving as support for more assertive decision-making regarding the diagnosis, as well as regarding other practices that should be adopted. However, in addition to the use of technology, it was also necessary to create differentiated care processes, given the need for asepsis of examination rooms before and after their use by patients with suspected COVID-19, together with the need to keep such patients separate from the other users of diagnostic centers.

### Operational impact

The growing concern with protecting patients and medical staff has affected the management of diagnostic centers such that, in practice, it is equivalent to operating two facilities in one. For example, imaging centers have a major limitation related to the equipment required. For example, it is not easy to move a CT scanner to a separate room. Therefore, many clinics segregated spaces, dedicating certain areas to the diagnosis/treatment of COVID-19. As a consequence, there was a decrease in medical productivity, because the teams, which had already been suffering from reductions and readjustments due to the drop in demand for examinations in general, had to adapt to following separate protocols for patients who were under suspicion of having the disease and for those who were not.

### Repressed demand

The developments described above give rise to another issue: the demand for the treatment of chronic diseases and elective procedures was repressed. Preoperative imaging examinations, for example, still need to be performed, because the underlying conditions did not simply disappear; such examinations were only postponed due to the fact that patients were exercising greater caution by not seeking medical care during the pandemic. The concern with a post-pandemic scarcity of health care services is also due to reductions in the human resources available. There is, therefore, an expectation of high demand in the post-pandemic period. Until then, there should be a gradual return that will present challenges, especially those involving the sizing of wards to care for patients with COVID-19 and for those with other diseases.

### Management of staffing

This is a serious time that requires us to make difficult decisions for the management of diagnostic centers. In relation to staffing, we have seen a movement toward short-term solutions such as reduced hours, wage cuts, and compulsory vacations. Leaders, more than ever, must rely on transparent communication to inform their decisions and motivations. This is the best way to avoid friction and promote a more empathetic approach, especially for the professionals directly affected by the changes and, above all, in cases such as dismissals. One major problem is related to the increased levels of anxiety and psychological stress during the pandemic. A study conducted by the University of Southern California, involving approximately 700 radiologists, indicated that 67% of those physicians reported an anxiety level of 7 on a scale of 10, with a mean of 6.72^(3)^. When asked what they were doing to deal with that anxiety, 63% reported trying to spend more time with their families, 57% reported trying to engage in physical activities, and many reported that they were not even trying to deal with it because they were “too busy with work”. One of the aggravating factors has to do with the explosion in the number of digital meetings through applications like Zoom, which take up a lot of the time of physicians. That survey highlighted other stressors for physicians: concerns about the health of their family members (reported by 71%); concerns about their own health (reported by 47%); and financial concerns (reported by 33%).

Another noteworthy issue is the impact that the management of staffing at diagnostic centers has had on the lives of female employees. There have been a number of studies of that topic, largely because of the position that women occupy in society, given that duties such as caring for children, the home, and the family in general often fall to them^(1)^. The impact is even greater if we consider that many women give up their careers because of those duties and that the productivity of those who remain may be affected. The American Association of Women in Radiology has provided additional information on the subject, reporting a 23% reduction in the number of articles in which the lead author was a woman, a 16% reduction in the number of articles with at least one female author, and a 16% drop in the overall representation of women in groups of authors^(4)^.

### Remote work and teleradiology

The pandemic has been a boon to the work-at-home model (aka telecommuting or remote work)*.* Once the precedent of remote work has been set, it will undoubtedly remain in place for a long time, even in the health care sector, where it has historically not been well accepted. Remote medicine has been clearly proven to be effective, especially in teleradiology, the use of which intensified during the pandemic^(5)^. The remote diagnostics modality proved useful not only to make it possible to provide care in times of social isolation but also to protect health care professionals from risk groups.

Efforts to maintain the workflow also need support. Therefore, it is important that the managers of diagnostic centers draw up long-term plans to potentiate the remote work carried out by health care professionals, with access redundancy, data security, and infrastructure improvement-all so that physicians have the means to work on a large scale, continuously and remotely.

## IN THE ABSENCE OF A RECIPE, WHAT CAN WE DO?

It is clear that the consequences of the pandemic on the management of diagnostic centers are numerous and no less challenging than were those on other sectors. There is no recipe for handling the situation assertively. However, we can cite the following recommendations made by the president of the American College of Radiology, which were developed in conjunction with large medical associations in the United States and can be adapted for use in Brazil^6)^:

**Engage to improve.** Diagnostic centers can mobilize and make an effort to develop best practices for the market. Therefore, it makes sense to foster ties with societies, associations, and similar groups, in order to compare experiences, as well as to strengthen discussions among market players and even government representatives to work on options that can keep the industry active with the lowest possible impact. It is always better to work together than to try to deal with the pandemic independently.

**Invest in innovation.** This is a favorable period to promote studies that advance the practice of digital radiology. Therefore, it is interesting to dedicate professionals exclusively to foment new technologies and innovation, especially in relation to the practical assessment of the use of artificial intelligence. Fostering innovation is essential at this moment in time and as a means of preparing for the future.

**Stimulus for research and education.** It is essential to promote the training of professionals, through training, seminars, or even free webinars. There are a number of measures that can be taken to increase the quality of work and further the specialization of the professional.

History indicates that other waves are yet to come, whether from the coronavirus or from other challenging diseases. We need to be careful and prepare for that. Market analysts and major media outlets have also suggested that the financial recovery at diagnostic centers will be slow worldwide, growth being projected only for late 2021 and early 2022. We are going through two concurrent crises: a health crisis and an economic crisis. From our perspective, the former needs to be remedied urgently, starting with vaccination, which will take time to reach the level of quality required. Once effective vaccines are made widely available, the “confidence” factor will enter the discussion, given that people will continue to be cautious about exposing themselves and resuming their routines. The fact is that our train has ground to a halt and will probably be slow to get back on track. However, that does not mean that we should stop stoking the boiler.
